# Postprandial Responses on Serum Metabolome to Milk and Yogurt Intake in Young and Older Men

**DOI:** 10.3389/fnut.2022.851931

**Published:** 2022-05-04

**Authors:** Jinyoung Kim, Carola Blaser, Reto Portmann, René Badertscher, Corinne Marmonier, Adeline Blot, Jérémie David, Helena Stoffers, Ueli von Ah, Ueli Bütikofer, Guy Vergères, Dominique Dardevet, Sergio Polakof

**Affiliations:** ^1^Unité de Nutrition Humaine (UNH), INRAE, Université Clermont Auvergne, Clermont-Ferrand, France; ^2^Agroscope, Bern, Switzerland; ^3^CNIEL, Paris, France; ^4^Centre Hospitalier Universitaire (CHU) Clermont Ferrand, CRNH Auvergne, Clermont-Ferrand, France

**Keywords:** milk, yogurt, biomarkers, metabolomics, human study

## Abstract

The identification and validation of biomarkers of food intake (BFIs) is a promising approach to develop more objective and complementary tools to the traditional dietary assessment methods. Concerning dairy, their evaluation in terms of intake is not simple, given the variety of existing foods, making it difficult to establish the association between specific dairy products consumption and the effects on human health, which is also dependent on the study population. Here, we aimed at identifying BFI of both milk (M) and yogurt (Y) in 14 healthy young (20–35 years) and 14 older (65–80 years). After a 3-week run-in period of dairy exclusion from the diet, the subjects acutely consumed 600 ml of M or Y. Metabolomics analyses were conducted on serum samples during the following 6 h (LC-MS and GC-MS). Several metabolites showing increased iAUC after milk or yogurt intake were considered as potential BFI, including lactose (M > Y, 2-fold), galactitol (M > Y, 1.5-fold), galactonate (M > Y, 1.2-fold), sphingosine-1-phosphate (M > Y from 2.1-fold), as well as an annotated disaccharide (Y > M, 3.6-fold). Delayed serum kinetics were also observed after Y compared to M intake lysine (+22 min), phenylalanine (+45 min), tyrosine (+30min), threonine (+38 min) 3-phenyllactic acid (+30 min), lactose (+30 min), galactitol (+45min) and galactonate (+30 min). The statistical significance of certain discriminant metabolites, such as sphingosine-1-phosphate and several free fatty acids, was not maintained in the older group. This could be related to the physiological modifications induced by aging, like dysregulated lipid metabolism, including delayed appearance of dodecanoic acid (+60 min) or altered postprandial appearance of myristic acid (+70% C_max_), 3-dehydroxycarnitine (−26% C_min_), decanoylcarnitine (−51% C_min_) and dodecanoylcarnitine (−40% C_min_). In conclusion, candidate BFI of milk or yogurt could be identified based on the modified postprandial response resulting from the fermentation of milk to yogurt. Moreover, population specificities (e.g., aging) should also be considered in future studies to obtain more accurate and specific BFI.

## Introduction

An accurate evaluation of the intake of specific foods and food groups is essential to establish reliable links between nutrition and health in the different human populations (1, 2). Unfortunately, in most nutritional studies, data are collected based on self-reporting food frequency questionnaires or 24 h-food intake recalls, which are limited by their subjective nature (1, 3). Metabolomics has become an essential analytical strategy for nutrition studies, given its high sensitivity to detect a wide range of low-weight molecules in biological samples with a single measurement. Because metabolites are closely associated with phenotype traits, metabolomics allows not only to identify and quantify metabolites but also provides valuable information on the physiological role of the identified markers. In particular, biomarkers of food intake (BFIs) belong to the class of dietary and health biomarkers and measure the intake of specific food groups, foods, or food components. They can thus be used to estimate recent or average intake of these entities (4). Therefore, their identification in nutrition research is of interest as it could provide more objective and accurate measures in addition to the assessment of food consumption (5). Combining the more classical assessment of dietary intake with robust, validated BFIs is therefore a promising next step toward improved assessments in nutritional studies (6).

Milk and dairy products build an important food group of human diet since the domestication of cattle and the relevance of these products has been further developed through traditional techniques including fermentation (7). At present, various types of dairy products are available and consumed worldwide, particularly in western countries (8), given that they vehicle an important variety of nutrients, providing proteins with a wide range of amino acids and micronutrients. These attributes, among others (e.g., high nutrient bioavailability, a functional food matrix, rich in shortfall nutrients), support the idea of achieving adequacy of dairy foods consumption as a global public health strategy, with the potential to help reduce global disease burden. In this sense, more than half of the European countries currently recommend 2–4 servings of dairy per day, including 150–200 mL of milk and 100–250 g yogurt per serving (9).

Beyond their significant contribution to nutritional intakes (8), the impact of dairy products on human health has also been widely investigated (10–12). However, these effects may vary depending on the type of dairy products consumed. For instance, the consumption of fermented dairy products (like yogurt or cheese, but not other dairy) has been associated with a beneficial impact on inflammation (13), while an inverse association between metabolic syndrome outcomes and dairy consumption has been shown for the full fat, but not the low-fat dairy foods (14, 15). Therefore, the development of more objective and precise tools able to measure specifically the intake of each type of dairy product is a crucial goal to establish reliable associations between specific dairy product intake and their effects on human health. During the last decade, numerous metabolomics-based studies proposed several molecules as new potential dairy BFI, including lactose-derived metabolites and odd-chain fatty acids (C15:0 and C17:0) (16–21). However, their validation faces several difficulties to be fully completed, including the overlap between the dairy products (21) and the human fluids metabolomes, the presence of some of these potential markers in other food groups, and their potential *in vivo* production (9, 17). Interestingly, both the impact of dairy consumption on health outcomes (22) and the evaluation of BFI overall (23), have been shown to be influenced by the study population specificities, including aging, highlighting the need for specific dairy product-specific BFI to be identified and characterized in both adults and elderly individuals (24).

We have conducted a randomized, controlled, crossover intervention study with milk and yogurt, in two age groups, one group composed of young adult men, the second of older adult men. After a 3-week run-in period during which the consumption of dairy and fermented products was drastically reduced, a milk or yogurt serving was acutely consumed in a crossover design. Untargeted (LC-MS) and targeted metabolomics (GC-MS) signatures in serum were assessed during the run-in and the postprandial period to identify dairy BFIs as well as to characterize their specificity regarding the ingested dairy product and the age group.

## Materials and Methods

### Study Population

A total of 28 healthy men participated in the study, 14 young adult men (YA, 20–35 years) and 14 older adult men (OA, 65–80 years) (Supplementary Figure 1). A sample size in the range of 10–15 subjects has been shown to give sufficient statistical power to identify regulated postprandial metabolites and potential BFIs (19, 25). Telephone interview and medical check, including blood tests, were performed to screen the participants. The inclusion and exclusion criteria of the participants have been described elsewhere (26). Briefly, healthy lactose-tolerant individuals who consumed dairy products regularly (2–4 portion/d) were recruited. Individuals with diseases or other features which may influence the study results were excluded.

### Study Design

**Figure 1:**
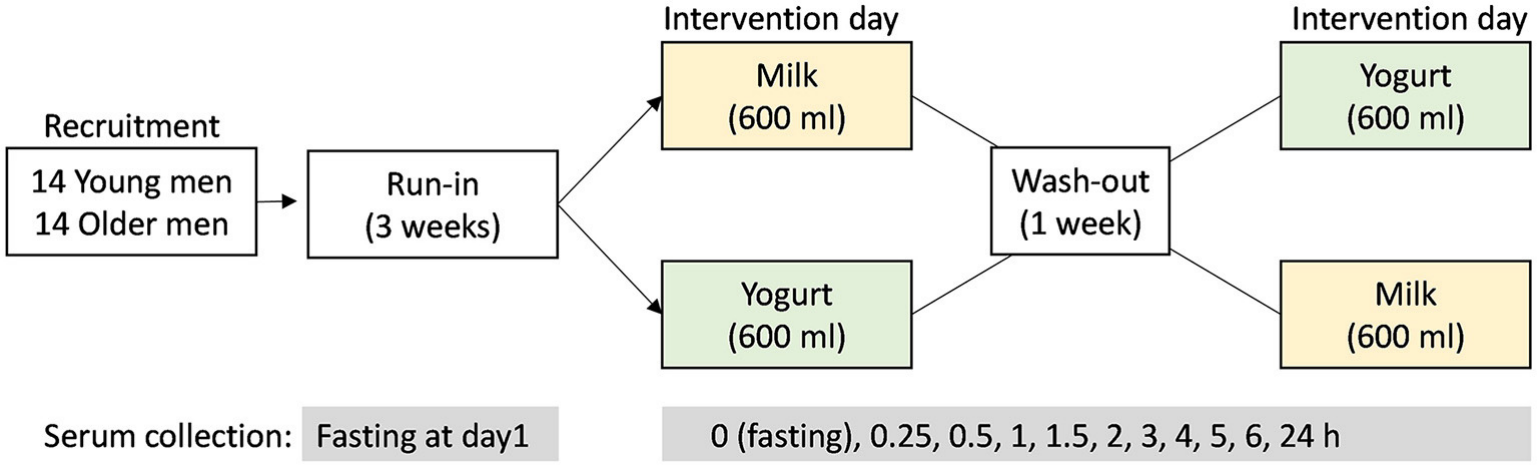
Study design of the randomized, controlled, crossover study. After the 3-week run-in period, the test products (milk or yogurt) were administrated in a randomized order. Serum samples were collected before (0 h) and up to 6 h after dairy product intake according to the defined intervals.

The current study is a randomized, controlled, crossover trial in YA and OA men (Figure 1). The trial was approved by the Ethical Committee of Personal Protection Ile de France IV (protocol code: 2017-A02879-44) and legal authorities and was registered at www.clinicaltrials.gov (NCT03500003). All study visits were conducted at the Human Nutrition Research Center of Auvergne (CRNH-A; Clermont-Ferrand, France) according to French law, between July 2018 and March 2019. All procedures were carried out in accordance with the guidelines laid down in the Declaration of Helsinki.

A 3-week run-in period was conducted before a crossover intervention consisting in the acute and single ingestion of a milk or yogurt serving. During the run-in period, the participants were requested to do not consume dairy foods and to limit the consumption of fermented non-dairy as described by Kim et al. (26) in order to maximize the postprandial metabolic signatures associated with the ingestion of milk or yogurt during the test day. On test day, the participants came to the research Center in the morning after an overnight fast and were randomly assigned to consume 600 ml of whole milk or yogurt. Both products were isocaloric (~65 kcal/100 g) and isoproteic (~3 g protein/100 g). The dose of 600 mL, although uncommon in normal dietary situations, was chosen to amplify the postprandial effect of dairy intake, thereby facilitating the identification of the metabolites and metabolic pathways that are most likely to change after normal chronic intake. Serum samples were collected at 10 time points during the 6 h postprandial challenge (0, 0.25, 0.5, 1, 1.5, 2, 3, 4, 5, 6 h). After the kinetics study, the participants consumed standardized lunch and dinner deprived of dairy and fermented foods. On the morning of the next day, participants came back to the research facilities and fasting serum was collected again. After 1 week of wash-out period, with no dairy product intake and reduced fermented food intake, a second postprandial challenge was conducted except that the alternative dairy product was ingested.

### Test Products

Non-commercial UHT whole milk and yogurt were tested. Both products were manufactured in parallel in the dairy research plant of Agroscope (Bern, Switzerland) using the same raw milk. For both products, fat content of the raw milk was standardized to 3.6% by centrifugation. The standardized raw milk was preheated to 65 ± 2 °C in a multipurpose heater with double jacket 90 L (SMKV, CH-3011 Berne) and homogenized in a two-stage process (160 ± 10 bar / 30 ± 5 bar) in a high-pressure homogenizer (Rannie, Typ LAB 12.50, DK-2620 Albertslund). UHT milk was produced by indirect heating the homogenized milk to 135 ± 1 °C in a UHT heater (JAG Jakob AG, CH-2555 Brügg) and immediate cooling down to 5 ± 2 °C. Exactly weighted portions of 150 g were filled into sterilized glass bottles using a Laminarflowbox (Spetec GmbH, D-85435 Erding). For yogurt production the homogenized milk was pasteurized at 92 ± 0.5 °C during 5 min and actively cooled to 43 °C [all processes in multipurpose heater with double jacket 90 L (SMKV, CH-3011 Berne)]. The pasteurized milk was inoculated with commercial mild starter culture YF-L 811 containing, according to the manufacturer information leaflet, *Lactobacillus delbrueckii* subsp. *bulgaricus* and *Streptococcus thermophilus* (Christian Hansen, DK-2970 Horsholm) according to specification. The inoculated milk was stirred and divided into batches by filling it into 12 l stainless steel buckets. The buckets were placed in an incubator with temperature control (BINDER TYP KB400, Binder GmbH, D-78532 Tuttlingen) and incubated at 43 °C until a pH value of 4.6 was reached. Then the samples were cooled to 5 °C and stored overnight. The pH measurement was performed in a 500 ml Schott flask in a separate water bath (42 °C) using Hamilton EasyFerm Bio 120 probes and recorded on Almemo 7.033 (Ahlborn, D-83607 Holzkirchen). The next day yogurt was stirred for 2 min at lowest intensity in a stirrer (Universal-Rührmaschine RN20 VL-2, Rotor Lips AG, CH-3661 Uetendorf). The whole batch of yogurt was divided into portions of 150 g, which were weighted exactly into commercial glass cups, covered with plastic seals (Winkler AG, CH-3510 Konolfingen) and stored at 5°C until shipping. The milk used in the study was provided by a local producer in Uettligen (CH-3043 Uettligen). Five batches of milk were produced to provide each study participant with fresh products for the acute challenge. For each batch of milk and yogurt stored at 4°C two samples were withdrawn for chemical and microbiological analyses before and after each clinical phase (about 9 weeks). The nutritional composition of raw milk, UHT milk and yogurt was assessed by MilcoScan Minor (Gerber Instruments, CH-8307 Effretikon, only raw milk) and in the Agroscope laboratory using accredited methods. The composition of the products is presented in Supplementary Table 1. The microbial composition of the products was measured using ISO methods 7889|IDF117:2003. Dilutions in the range from −2 to −6 were done and plated using a spiral plater (EddyJet, IUL Instruments) and evaluated using an automated colony counter (SpereFlash, IUL Instruments).

### Metabolomic Analyses

#### Untargeted LC-MS

The preparation and the measurement of serum samples by LC-MS followed the same method as described previously by Pimentel et al. (19). Briefly, 50 μl serum samples were mixed with 150 μl of acetonitrile solution with 1% of formic acid to precipitate proteins. Phospholipids were removed from the mixture with a filter membrane (Phree®, Phenomenex Inc., Torrance, California, USA). Milk and yogurt samples were prepared in a similar way as for serum. Thus, 0.3 g of product samples were weighed and three volumes of acetonitrile solution containing 1% of formic acid were added. The mixtures were centrifuged (12,000 g at 4 °C, 15 min) and the supernatant was filtered (0.22 μm regenerated cellulose, WhatmanTM UnifloTM 13/0.2 RC, and Phree plate). Four μl of the filtrates (serum or dairy product) were injected in the LC-MS.

The raw data was treated with pre-processing steps, including retention time alignment, peak-picking, deconvolution, and normalization with default settings (default automatic sensitivity and without minimum peak width) using Progenesis QI (v.2.3.6198.24128, Non-Linear Dynamics Ltd., Newcastle upon Tyne, United Kingdom). Signal drift correction was performed with R (v.4.0.3; R Foundation for Statistical Computing, Vienna, Austria) via the QC-based robust locally estimated scatterplot smoothing signal (LOESS) correction method (27). Metabolites with poor repeatability (detected in <50% of the QC samples and a relative standard deviation (RSD) >30% in the QC samples) were deleted. Metabolites with poor repeatability in samples (<80% in all groups) were also removed. Also, features that had a median in the QC samples below three times the median calculated for the blanks were excluded.

#### Targeted and Untargeted GC-MS

Using an internal GC-MS database, 14 metabolites were targeted in this study and additionally identified using retention indices (RI) (see Table 1). An additional 3 metabolites with significant differences were identified in the untargeted mode. Using the NIST database, two of these were identified at level 3 (octadecenoic acid and octadecadienoic acid). However, because the position of the double bonds, as well as the cis/trans isomerism could not be determined, these metabolites could not be identified at level 1.

**Table 1:** List of identified discriminant blood metabolites derived from LC-MS and GC-MS analyses with significant differences in their iAUC, C_max_ (C_min_), or t_max_ (t_min_) [median (IQR)] either by age, product, or their interaction after the intake of milk or yogurt in young and older men.

							**Young men**		**Older men**		**FDR /** ***P*****-value (Wald test)**^**a**^			
**Metabolite**	**Mass / Quantifier**	**RT / RI**	**ID^**f**^**	**Product^**c**^**	**Run-in^**d**^**		**Milk**	**Yogurt**	**Milk**	**Yogurt**	**Age**	**Product**	**Interaction**	**24 h^**e**^**
**Lipids, fatty acids and derivatives**														
2-Hydroxybutyric acid	131	RI: 1,120	1	-	N.S.	iAUC^g^	1.2 (0.13, 1.47)*^*p*^*	1.61 (1.34, 1.96)^p^*	1.68 (1.01, 2.42)*^*p*^*#	1.49 (1.14, 2.07)*^*p*^*	*0.312*	*0.125*	***0.024***	↑ in OA (M)
						C_max_^h^	0.41 (0.29, 0.62)	0.60 (0.54, 0.68)	0.69 (0.57, 0.75)	0.56 (0.45, 0.74)	*0.181*	*0.533*	***0.049***	
						t_max_^i^	5.00 (2.38, 5.75)	5.00 (1.25, 6.00)	5.00 (4.25, 6.00)	5.00 (1.88, 6.00)	*0.614*	*0.951*	*0.922*	
Decanoic acid	229	RI 1,454	1	-	↓ in OA	iAUC	4.37 (3, 5.82)*^*p*^*	4.96 (4.35, 6.05)*^*p*^*	5.62 (4.65, 6.2)*^*p*^*	6.44 (5.55, 6.8)^p^*	*0.128*	***0.006***	*0.336*	↑ in OA (Y)
						C_max_	1.43 (1.10, 2.32)	1.66 (1.43, 1.98)	1.65 (1.51, 1.94)	1.70 (1.47, 1.90)	*0.616*	*0.685*	*0.868*	
						t_max_	3.50 (0.62, 4.00)	3.00 (2.00, 4.00)	3.50 (3.00, 4.00)	3.00 (2.00, 4.00)	*0.770*	*0.240*	*0.586*	
Dodecanoic acid	257	RI: 1,648	1	-	N.S.	iAUC	1.97 (0, 3.69)	3.36 (2.19, 3.93)*^*p*^*	3.07 (2.25, 3.64)*^*p*^*	4.04 (2.13, 4.8)*^*p*^*	*0.174*	***0.003***	*0.979*	↓ in YA (M)
						C_max_	0.98 (0.62, 1.62)	1.18 (0.78, 1.57)	1.57 (1.50, 1.98)#	1.49 (1.08, 1.72)	***0.031***	*0.555*	*0.189*	
						t_max_	4.50 (4.00, 5.00)	3.50 (2.25, 4.00)	5.00 (4.25, 5.00)	5.00 (4.00, 5.00)#	***0.027***	***0.023***	*0.111*	
Myristic acid	285	RI: 1,842	1	-	N.S.	iAUC	0.78 (−0.4, 1.39)	1.11 (−0.09, 2.07)	1.71 (0.71, 2.24)*^*p*^*	1.81 (0.59, 3.04)*^*p*^*	*0.100*	*0.181*	*0.618*	↓ in YA (M)
						C_max_	0.60 (0.39, 0.97)	0.71 (0.43, 0.86)	1.12 (0.96, 1.27)#	1.10 (0.76, 1.28)	***0.006***	*0.606*	*0.690*	
						t_max_	5.00 (4.00, 5.00)	4.00 (3.00, 5.00)	5.00 (4.25, 5.00)	5.00 (4.00, 5.75)	*0.082*	*0.226*	*0.121*	
Glycocholic acid	465.3081 (n)	RT: 9.17	1	-	N.S.	iAUC	2.4 (0.49, 5.76)^p^	4.92 (3.82, 6.45)^p^*	3.15 (1.09, 5.04)^p^	2.08 (0.58, 4.82)#	0.229	0.194	**0.025**	↓ in OA (M,Y)
						C_max_	2.25 (1.25, 3.49)	2.51 (1.73, 3.57)	1.43 (1.30, 1.87)	1.46 (1.05, 2.68)	**0.088**	0.438	0.355	
						t_max_	2.00 (0.75, 2.75)	2.00 (1.50, 3.00)	1.00 (0.50, 2.75)	1.50 (1.00, 2.00)	0.318	0.323	0.816	
Sphingosine-1-phosphate^b^	379.2481 (n)	RT: 11.6	1	-	N.S.	iAUC	0.93 (−0.14, 1.77)*^*p*^*	−0.41 (−1.46, 0.04)*	0.34 (-0.85, 0.99)	−0.68 (−1.11, −0.11)	0.617	**0.051**	0.675	↓ in YA (Y)
3-Dehydroxycarnitine	146.1173 (m/z)	RT: 1.04	1	M < Y	N.S.	iAUC	−0.24 (−0.51, 0.05)	−0.92 (−1.26, −0.39)^p^*	−1.09 (−1.24,−0.57)*^*p*^*#	−1.23 (−1.93, −0.88)*^*p*^*	**0.060**	**0.039**	0.600	N.S.
						C_min_	−0.22 (-0.24,−0.16)	−0.34 (-0.41,−0.21)*	−0.32 (-0.45,−0.24)#	−0.44 (-0.55,−0.27)	**0.029**	**0.001**	0.212	
						t_max_	1.50 (1.12, 2.75)	1.75 (1.50, 4.75)	1.50 (1.12, 2.00)	2.00 (1.50, 3.00)	0.833	0.248	0.569	
Decanoylcarnitine	316.2476 (m/z)	RT: 8.86	1	-	N.S.	iAUC	−1.05 (−2.49, −0.22)	−3.42 (−4.14, −1.22)*^*p*^*	−5.4 (−7.54, −1.57)*^*p*^*#	−5.05 (−6.73, −3.53)*^*p*^*#	**0.065**	0.162	0.675	
						C_min_	−0.37 (−0.77, −0.25)	–.96 (−1.34, −0.47)	−1.42 (−1.66, −0.75)#	−1.34 (−1.87, −1.21)#	**0.004**	**0.081**	0.446	N.S.
						t_min_	1.25 (1.00, 1.50)	2.00 (1.12, 4.25)	1.50 (1.50, 2.00)	2.00 (1.62, 2.75)	0.340	**0.011**	0.460	
Dodecanoylcarnitine	344.2787 (m/z)	RT: 10.08	1	-	N.S.	iAUC	−0.7 (-1.48, 0.75)	−2.2 (-3.08,−0.21)*^*p*^*	−1.91 (-7.08,−0.69)*^*p*^*	−2.6 (-5.28,−1.88)*^*p*^*	0.293	0.109	0.919	↓ in OA (Y)
						C_min_	−0.38 (−0.96, −0.16)	−0.8 (−1.18, −0.34)	−1 (−1.79,−0.45)#	−0.93 (−1.62, −0.8)	**0.059**	**0.023**	0.358	
						t_min_	1.50 (0.62, 4.00)	2.00 (1.25, 4.50)	1.75 (1.00, 2.00)	1.50 (1.12, 2.00)	0.523	0.493	0.365	
Octadecenoic acid	339	RI:2214	3	-	↑in YA	iAUC	−2.48 (−3.58, −1.12)*^*p*^*	−1.86 (−2.25, −0.7)*^*p*^*	−1.35 (−2.03,−0.66)*^*p*^*	−1.23 (−2.25, −0.12)*^*p*^*	*0.157*	*0.244*	*0.158*	↓ in YA (M, Y)
					↑in OA	C_min_	−0.82 (−1.05, −0.54)	−0.58 (−0.85, −0.42)	−0.58 (−0.75, −0.37)	−0.49 (−0.66, −0.31)	*0.269*	***0.032***	*0.213*	↓ in OA (M, Y)
						t_min_	2.00 (1.50, 2.00)	2.00 (1.50, 3.00)	2.00 (1.62, 2.00)	1.75 (1.50, 2.00)	*0.654*	*0.788*	*0.745*	
Octadecadienoic acid	337	RI:2209	3	-	↑ in YA	iAUC	−2.68 (−4.3, −1.91)*^*p*^*	−2.04 (−2.69, −0.97)*^*p*^*	−1.6 (-2.19, −1.05)*^*p*^*	−1.58 (−2.28, −0.62)*^*p*^*	*0.113*	*0.155*	*0.093*	↓ in YA (M, Y)
					↑ in OA	C_min_	−0.85 (−1.21, −0.57)	−0.62 (−0.87, −0.39)*	−0.54 (-0.63, −0.42)	−0.53 (−0.62, −0.35)	*0.177*	***0.041***	*0.109*	↓ in OA (M)
						t_min_	2.00 (1.50, 2.00)	2.00 (1.50, 2.75)	2.00 (1.50, 3.00)	2.00 (1.50, 3.00)	*0.657*	*0.598*	*0.860*	
**Amino acids and derivates**														
Aspartic acid	232	RI: 1,509	1		N.S.	iAUC	1.42 (1.06, 1.96)*^*p*^*	0.88 (−0.09, 1.54)*^*p*^*	0.92 (0.53, 1.74)*^*p*^*	0.63 (−0.38, 1.21)	*0.221*	***0.020***	*0.805*	↑ in YA (M)
						C_max_	1.75 (1.00, 2.75)	1.75 (1.00, 2.00)	3.00 (1.62, 5.00)	1.00 (0.50, 3.75)	*0.515*	*0.185*	*0.179*	
						t_max_	0.79 (0.64, 1.11)	0.50 (0.40, 0.85)	0.57 (0.39, 0.81)	0.57 (0.30, 0.77)	*0.331*	*0.115*	*0.484*	
Lysine	146.1053 (n)	RT: 0.88		-	N.S.	iAUC	1.14 (0.78, 1.41)*^*p*^*	0.88 (0.37, 1.19)*^*p*^*	0.9 (0.68, 1.31)*^*p*^*	0.62 (0.15, 0.95)	0.689	0.355	0.908	N.S.
						C_max_	0.41 (0.28, 0.50)	0.51 (0.45, 0.63)*	0.49 (0.39, 0.61)	0.38 (0.30, 0.49)#	0.490	0.730	**0.002**	
						t_max_	0.50 (0.50, 1.00)	1.25 (1.00, 1.50)	1.00 (0.50, 1.38)	1.00 (1.00, 1.50)	0.514	**0.013**	0.340	
Ornithine	132.0897 (n)	RT: 0.88	1	-	N.S.	iAUC	0.71 (0.37, 0.9)*^*p*^*	0.37 (−0.06, 0.75)	0.82 (0.31, 1.24)*^*p*^*	0.14 (−0.45, 0.55)	0.965	0.210	0.664	↓ in YA (M)
						C_max_	0.24 (0.18, 0.29)	0.24 (0.13, 0.35)	0.35 (0.24, 0.43)#	0.14 (0.12, 0.25)*	0.853	**0.023**	**0.010**	↓ in OA (Y)
						t_max_	1.75 (0.62, 2.75)	1.25 (1.00, 2.00)	3.00 (1.25, 3.00)	1.75 (0.50, 2.00)	0.264	0.152	0.202	
Phenylalanine	165.0788 (n)	RT: 3.14	1	M < Y	↓ in OA	iAUC	0.3 (-0.02, 0.92)*^*p*^*	0.26 (-0.25, 0.82)	0.23 (-0.54, 0.79)	0.33 (0.09, 0.68)	0.922	0.892	0.768	N.S.
						C_max_	0.32 (0.25, 0.38)	0.36 (0.32, 0.42)	0.29 (0.22, 0.42)	0.32 (0.25, 0.36)	0.212	0.395	0.233	
						t_max_	0.50 (0.31, 0.50)	1.00 (1.00, 1.50)*	0.75 (0.50, 1.38)#	1.75 (1.12, 2.00)*	**0.007**	**<** **0.001**	0.727	
Proline	115.0632 (n)	RT: 1.04	1	M < Y	↓ in YA	iAUC	1.34 (1.04, 1.72)*^*p*^*	1.45 (0.71, 1.69)*^*p*^*	1.35 (1.23, 1.57)*^*p*^*	1.56 (1.05, 1.98)*^*p*^*	0.977	0.804	0.832	N.S.
					↓ in OA	C_max_	0.41 (0.36, 0.42)	0.61 (0.50, 0.71)*	0.46 (0.40, 0.53)	0.50 (0.41, 0.58)	0.703	**0.001**	**0.024**	
						t_max_	1.50 (1.00, 2.00)	1.50 (1.50, 2.00)	1.25 (1.00, 2.75)	2.00 (1.62, 3.00)	0.375	0.235	0.232	
Tyrosine	218	RI: 1,931	1	-	N.S.	iAUC	0.41 (0.29, 0.75)*^*p*^*	0.6 (0.43, 0.99)^p^*	0.71 (0.51, 0.97)*^*p*^*	1.04 (0.53, 1.27)*^*p*^*	***0.035***	*0.094*	*0.870*	↑ in YA (Y)
						C_max_	0.35 (0.22, 0.40)	0.46 (0.35, 0.59)*	0.34 (0.24, 0.47)	0.41 (0.37, 0.49)*	*0.692*	***0.001***	*0.567*	↑ in OA (M)
						t_max_	1.00 (0.62, 1.50)	1.50 (1.12, 1.88)*	1.00 (1.00, 2.75)	1.50 (1.50, 1.50)	*0.429*	***0.027***	*0.444*	
N-Methyl proline	130.0860 (m/z)	RT: 0.88	1	-	N.S.	iAUC	1 (0.74, 1.45)*^*p*^*	0.89 (0.33, 1.2)*^*p*^*	1 (0.59, 1.25)*^*p*^*	0.81 (0.29, 1.07)	0.837	0.459	0.897	N.S.
						C_max_	0.42 (0.27, 0.51)	0.50 (0.40, 0.70)	0.48 (0.37, 0.59)	0.37 (0.31, 0.54)	0.547	0.797	**0.016**	
						t_max_	0.50 (0.50, 1.38)	1.25 (1.00, 1.50)	0.75 (0.50, 1.00)	1.25 (1.00, 1.50)	0.994	**0.005**	0.610	
Threonine	119.0581 (n)	RT: 0.95	1	-	N.S.	iAUC	0.44 (0.32, 0.93)*^*p*^*	0.62 (−0.48, 0.83)	−0.05 (−0.32, 0.79)	−0.06 (−0.84, 0.57)	0.201	0.699	0.908	↓ in OA (M,Y)
						C_max_	0.31 (0.24, 0.38)	0.39 (0.27, 0.48)	0.34 (0.20, 0.37)	0.24 (0.13, 0.30)#	**0.053**	0.759	**0.096**	
						t_max_	1.00 (0.50, 1.00)	1.50 (1.00, 2.00)	1.00 (0.62, 1.88)	1.75 (1.00, 2.00)	0.320	**0.013**	0.454	
3-Phenyllactic acid	193	RI: 1,580	1	M < Y	N.S.	iAUC	−0.61 (−0.92, −0.04)*^*p*^*	0.99 (0.67, 1.38)^p^*	−0.28 (−0.63, 0)*^*p*^*	1.09 (0.71, 1.37)^p^*	*0.474*	***<** **0.001***	*0.847*	↑ in OA (Y)
						C_max_	0.07 (0.04, 0.16)	0.50 (0.39, 0.55)*	0.09 (0.05, 0.19)	0.40 (0.32, 0.45)*	*0.737*	***<** **0.001***	*0.383*	
						t_max_	0.75 (0.31, 2.00)	1.50 (1.50, 2.00)	1.25 (1.00, 5.25)	1.50 (1.50, 3.00)	*0.245*	***0.040***	*0.518*	
3-Methylhistidine	96	RI: 1,860	2	-	↑ in YA	iAUC	−0.13 (−0.6, 0.57)	−0.38 (−3.43, 0.8)	−1.46 (-2.94, −0.71)*^*p*^*#	−0.23 (-1.13, 0.12)	*0.253*	*0.488*	***0.031***	N.S.
					↑ in OA									
**Carbohydrates and derivatives**														
Lactose	204	RI: 2,666	1	M < Y^l^	↓ in YA	iAUC	5.35 (3.25, 6.59)*^*p*^*	2.24 (1.87, 2.92)^p^*	6 (4.27, 13.31)*^*p*^*	3.05 (2.57, 4.63)^p^*#	***0.034***	***<** **0.001***	*0.278*	↑ in YA (M, Y)
					↓ in OA	C_max_	1.36 (0.86, 1.82)	0.62 (0.58, 0.87)*	1.93 (1.00, 3.22)	0.84 (0.70, 1.33)*	*0.064*	***<** **0.001***	*0.658*	↑ in OA (M, Y)
						t_max_	1.50 (1.50, 2.00)	3.00 (2.00, 3.00)*	3.00 (2.00, 3.75)#	2.50 (2.00, 4.00)	***0.036***	***0.004***	***0.015***	
Galactose	319	RI: 1,874	1	M < Y^l^	N.S.	iAUC	6.92 (4.39, 7.16)*^*p*^*	6.83 (5.38, 7.78)*^*p*^*	9.29 (5.17, 12.95)*^*p*^*	8.43 (5.51, 9.36)*^*p*^*	*0.206*	*0.817*	*0.441*	N.S.
						C_max_	4.94 (3.95, 6.93)	4.81 (2.80, 5.62)*	6.16 (3.78, 8.71)	3.62 (2.55, 5.03)*	*0.867*	***<** **0.001***	*0.199*	
						t_max_	1.00 (1.00, 1.00)	1.00 (0.62, 1.50)	1.00 (0.62, 1.50)	1.25 (1.00, 1.50)	*0.472*	*0.252*	*0.736*	
Galactitol	319	RI: 1,925	1	-	↓ in YA	iAUC	3.75 (2.67, 4.26)*^*p*^*	2.69 (2.15, 3.12)^p^*	3.98 (1.84, 4.3)*^*p*^*	2.62 (1.92, 2.91)*^*p*^*	*0.517*	***0.001***	*0.946*	↑ in YA (Y)
						C_max_	1.29 (1.08, 1.35)	0.83 (0.72, 1.03)*	1.08 (0.75, 1.42)	0.79 (0.62, 0.97)*	*0.323*	***<** **0.001***	*0.575*	↑ in OA (M,Y)
						t_max_	2.00 (2.00, 2.00)	3.00 (2.00, 4.00)*	2.50 (1.62, 3.00)	3.00 (2.25, 4.00)*	*0.401*	***<** **0.001***	*0.520*	
Galactonate	292	RI: 1,979	1	M < Y	↓ in OA	iAUC	5.89 (4.86, 8.17)*^*p*^*	4.47 (4.03, 5.17)^p^*	6.3 (5.63, 8.92)*^*p*^*	5.47 (3.5, 6.73)^p^*	*0.749*	***<** **0.001***	*0.544*	↑ in YA (M, Y) ↑ in OA (M, Y)
						C_max_	1.62 (1.33, 2.46)	1.32 (1.25, 1.70)*	2.13 (1.57, 2.64)	1.60 (1.08, 2.02)*	*0.439*	***<** **0.001***	*0.821*	
						t_max_	3.00 (2.00, 3.75)	4.00 (3.00, 4.00)*	4.00 (3.00, 4.00)	4.00 (4.00, 5.00)*#	***0.005***	***<** **0.001***	*0.347*	
X0590	361	RI:2815	3	M < Y	N.S.	iAUC	4.2 (3.6, 5.76)*^*p*^*	16.06 (12.96, 23.52)^p^*	4.78 (3.5, 5.91)*^*p*^*	14.63 (9.66, 17.88)^p^*	*0.516*	***<** **0.001***	*0.334*	↑ in YA (M, Y)
						C_max_	1.14 (1.04, 1.33)	5.02 (3.40, 6.37)*	1.36 (1.07, 1.67)	4.06 (3.14, 5.99)*	*0.867*	***<** **0.001***	*0.117*	↑ in OA (M, Y)
						t_max_	3.00 (2.25, 3.75)	3.00 (3.00, 3.75)	4.00 (3.25, 4.00)	4.00 (4.00, 4.00)#	***0.004***	*0.917*	*0.755*	
Glycerol	205	RI: 1,264	1	-	N.S.	iAUC	−1.45 (−2.84, −0.71)*^*p*^*	−0.81 (−1.65, −0.18)*^*p*^*	−1.21 (−2.56,−0.39)*^*p*^*	−1.17 (−2.08, −0.09)*^*p*^*	*0.743*	*0.078*	*0.169*	↓ in YA (M)
						C_min_	−0.56 (−0.76, −0.34)	−0.37 (−0.66, −0.18)	−0.47 (-0.69, −0.33)	−0.37 (−0.72, −0.28)	*0.918*	***0.029***	*0.350*	
						t_min_	1.25 (1.00, 1.50)	1.75 (1.00, 2.75)	1.50 (1.12, 2.00)	1.50 (1.50, 1.88)	*0.513*	*0.200*	*0.234*	
**Other organic compounds**														
Epinephrine	166.0860 (m/z)	RT: 1.27	1	M < Y	↓ in OA	iAUC	0.47 (0.16, 0.65)*^*p*^*	0.18 (0.01, 0.59)	0.2 (-0.26, 0.85)	0.01 (−0.39, 0.48)	0.557	0.494	0.938	↓ in OA (Y)
						C_max_	0.33 (0.27, 0.37)	0.35 (0.30, 0.46)	0.34 (0.24, 0.51)	0.25 (0.16, 0.33)	0.148	0.347	0.121	
						t_max_	0.50 (0.50, 0.50)	1.50 (1.50, 1.50)*	0.50 (0.50, 1.00)	1.00 (0.62, 2.00)	0.628	**<** **0.001**	**0.050**	
Citrate	273	RI: 1,801	1	-	N.S.	iAUC	0.59 (0.38, 1.58)*^*p*^*	0.45 (-0.18, 1.42)	0.21 (−0.01, 0.81)	0.75 (−0.14, 1.52)*^*p*^*	*0.578*	*0.891*	*0.111*	N.S.
						C_max_	0.33 (0.27, 0.49)	0.38 (0.17, 0.55)	0.22 (0.18, 0.30)	0.33 (0.21, 0.48)	*0.192*	*0.498*	*0.104*	
						t_max_	1.00 (0.50, 1.50)	1.50 (0.75, 2.00)	1.00 (0.31, 1.00)	1.25 (1.00, 1.88)	*0.766*	***0.003***	*0.753*	
Isocitric acid^b^	192.0275 (n)	RT: 1.23	1	M > Y	↑ in YA ↑ in OA	iAUC	0.54 (-0.19, 0.93)	0.74 (0.33, 1.03)	−0.29 (−0.71, 0.3)	−0.13 (−0.57, 0.39)	**0.001**	0.415	0.852	↓ in YA (Y) ↓ in OA (M,Y)
Inosine^b^	268.0803 (n)	RT: 2.64	1	-	↓ in YA ↓ in OA	iAUC	0.54 (-0.19, 0.93)	0.74 (0.33, 1.03)*^*p*^*	−0.29 (−0.71, 0.3)#	−0.13 (−0.57, 0.39)#	**0.045**	0.684	0.937	N.S.

^p^Significant postprandial response, iAUC ≠ 0 by Wilcoxon signed-rank test (P-value < 0.05). *Significant difference between milk and yogurt (product effect, P-value < 0.05) by paired Wilcoxon signed-rank test; # Significant difference between groups (age effect, P-value < 0.05) by Wilcoxon signed-rank test; ^a^P-value calculated by Wald Chi-Squared test, statistical significance is indicated in bold when P < 0.05 for targeted GC-MS (italic) and when FDR < 0.10 for untargeted LC-MS after FDR correction (normal font). ^b^No kinetic parameters were calculated; ^c^comparison of the concentration between in milk and in yogurt by Wilcoxon signed-rank test when the metabolite was detected, M < Y: higher concentration in yogurt than milk, M > Y: higher concentration in milk than yogurt, M = Y: no significant differences between milk and yogurt concentration, -: not detected, P < 0.05; ^d^comparison the fasting level of metabolites between before and after the run-in period, ↑: significantly increased during the run-in period, ↓: significantly decreased during the run-in period, P < 0.05; ^e^comparison between the fasting levels of baseline at postprandial challenge (0 h) and after 24 h (24 h) by Wilcoxon signed-rank test, ↑: significantly increased 24 h after the intake of dairy, ↓: significantly decreased 24 h after intake of dairy, P < 0.05; ID level, identification level; M, milk; OA, older adult; RI, Retention index (for GC-MS); RT, Retention time (for LC-MS); Y, yogurt; YA, young adult. ^f^Level of identification: 1 identified by comparison to a pure reference based on spectral data, 2 without chemical standards, based on spectral data, 3 putatively characterized compound classes; ^g^iAUC incremental area under curve [A.U. * 6 h]; ^h^Cmax highest concentration [A.U]; ^i^tmax time at Cmax; ^j^Cmin lowest concentration [A.U]; ^k^tmin time at Cmin, ^l^data from Supplementary Table 1.

Preparation of the serum samples and their measurement by GC-MS followed the method described previously by Trimigno et al. (17). Briefly, a two-step derivatization, consisting of methoximation and silylation, was applied to 100 μL of serum sample and the samples were analyzed on a GC-MS 7890B/MS5977A (Agilent Technologies, Santa Clara, CA, USA) with a CombiPAL autosampler (CTC-Analytics AG, Zwingen, Switzerland) and a DB-5 ms fused silica capillary column (60 m, 0.25 mm i.d., 0.25 μm film thickness, Agilent Technologies, Basel, Switzerland). Post-processing of the data was performed using Agilent MassHunter software (V.10) with the Unknown and Massprofiler modules. Signal drift correction was also applied (27) and the filtration of metabolites was conducted with the same criteria as for LC-MS.

### Statistical Analyses

Non-parametric robust statistical tests were used in this study due to many variables not showing a normal distribution. Descriptive analysis, including median with interquartile range (IQR), was performed for all variables. All data was processed in the R environment (4.0.3). Baseline differences between the age groups were assessed for all parameters by comparing the samples at time point 0 of the YA and OA groups by Wilcoxon signed-rank test. A *P*-value < 0.05 was considered statistically significant.

To identify features derived from the untargeted LC-MS and targeted GC-MS that presented a significant postprandial time response (at least one time point) after intake of either milk or yogurt, nonparametric analysis of longitudinal data with ld.f1 function was used (nparLD R package, *P* < 0.05) (28). For features with a significant time response, the incremental area under the curve (iAUC) for the 6 h postprandial phase was further calculated by the MESS package (version 0.3.2). A Wilcoxon signed-rank test (*P* < 0.05) was then applied to test whether the iAUC of each feature after milk or yogurt intake in the YA or OA group was significantly different from 0 to confirm it as a postprandial-responding variable. A feature was considered to demonstrate a postprandial response if it was significant (*P* < 0.05) for both statistical tests (ld.f1 and Wilcoxon signed-rank test) in at least one of the four groups (YA-M, YA-Y, OA-M, OA-Y). Exploratory analyses of the postprandial-responding features derived from the LC-MS were conducted by clustering analyses (R package pheatmap v1.0.12) (29) after the normalization to the maximum value of each feature. The postprandial-responding variables were then tested using the f1.ld.f1 function of the nparLD package (Wald Chi-Squared test, *P* < 0.05) to investigate the effect of age, product, and their interaction during the 6 h period following the intake of the dairy products. Features were considered as significant after Benjamini-Hochberg (30). FDR correction for the untargeted LC-MS dataset (*P* < 0.05 and FDR < 0.1) and without FDR correction for the targeted GC-MS dataset (*P* < 0.05). The features showing significant differences in age, product, and/or an interaction effect, were further evaluated with a Wilcoxon signed-rank test to identify which of them responding differently after milk and yogurt intake in the YA and OA groups separately (paired, *P* < 0.05) as well as which of them showed different postprandial responses by age group separately (non-paired, *P* < 0.05).

For those features presenting significant differences in age, product, and/or interaction during the postprandial phase, their fasting values at 0 h were compared to the values during the run-in period using a Wilcoxon signed-rank test (paired, *P* < 0.05). To evaluate whether the significant postprandial features were maintained after 24h, comparison between the fasting levels of baseline at postprandial challenge (0h) and after 24 h were also made using the Wilcoxon signed-rank test (paired, *P* < 0.05). In addition, these variables (metabolites) were targeted in the metabolome of milk and yogurt to verify their presence in the dairy products. Concerning dairy products two samples of UHT milk and yogurt derived from five batches of raw milk were available. Significant differences in the concentrations of the features between the two dairy products were evaluated by the Wilcoxon signed-rank test (non-paired, *P* < 0.05). No differences were noticed between the samples at the beginning and at the end of each clinical phase (about 9 weeks, data not shown).

### Identification of Discriminant Features

Discriminating features derived from the untargeted metabolomics LC-MS analyses were subjected to identification as described in Pimentel et al. (19). Shortly, the Human Metabolome Database (31) and the National Institute of Standards and Technology database (NIST v14) were used to identify the features. The following two orthogonal criteria were used to consider a level 1 identification of the metabolites: (i) mass accuracy: ∓ 5 ppm; (ii) retention time of feature in sample compared to retention time of pure standard: ∓ 10% (32, 33). A targeted approach was used for the identification of 17 discriminant features from GC-MS, using an in-house reference compound library, retention indices (RI), and spectral data as described in Münger et al. (16).

### Kinetic Variables Calculation

The postprandial metabolomic features that showed significant postprandial responses (see **Figure 3**, clusters 1 and 7) were selected for kinetic analyses. The maximum concentration (C_max_) and the time to reach C_max_ (t_max_) were analyzed by non-compartment methods using the ncappc package implemented in R (v 4.0.3.) (34). The minimum concentration (C_min_) and time to reach C_min_ (t_min_) were calculated in case of metabolites that showed negative postprandial responses.

## Results

### Baseline Characteristics of Participants at the Beginning of the Postprandial Challenge

The median age of the YA group was 27.5 years (IQR 25.0, 31.0) and that of OA was 69.0 years (66.0, 71.0). Although the weight of the YA group was higher than the OA group, their body mass index (BMI) was not significantly different because participants in the YA group also are taller: 1.79 m (1.73, 1.85) vs. 1.73 m (1.69, 1.74), *P* = 0.009.

### Dairy Products Characterization

The chemical composition of the products is shown in Supplementary Table 1. The complete absence of D-lactate indicated that no metabolic active *L*. *bulgaricus* was present. This could be confirmed by the microbial composition analysis of the yogurt according to ISO 7889|IDF 117:2003. Using an extended range of 10-fold dilutions (−2 to −6, covering 10^2^ to 10^9^ cfu/g) no *L. delbrueckii* subsp. *bulgaricus* could be determined in the final yogurt whereas 2.3x10^7^ cfu/g *S. thermophilus* could be detected. Microscopically, the dominance of *S. thermophilus* could be seen in the 1,000 x concentrated culture (Supplementary Figure 2, panel A) as well as in the final yogurt (Supplementary Figure 2, panel B). For a mild acidifying yogurt, the absence or reduction of the metabolic activity of *L. delbrueckii* subsp. *bulgaricus* is a desired trait. With the current requirements for yogurt denomination the requirements are still met since *L. delbrueckii* subsp. *bulgaricus* is present although at very low levels.

**Figure 2:**
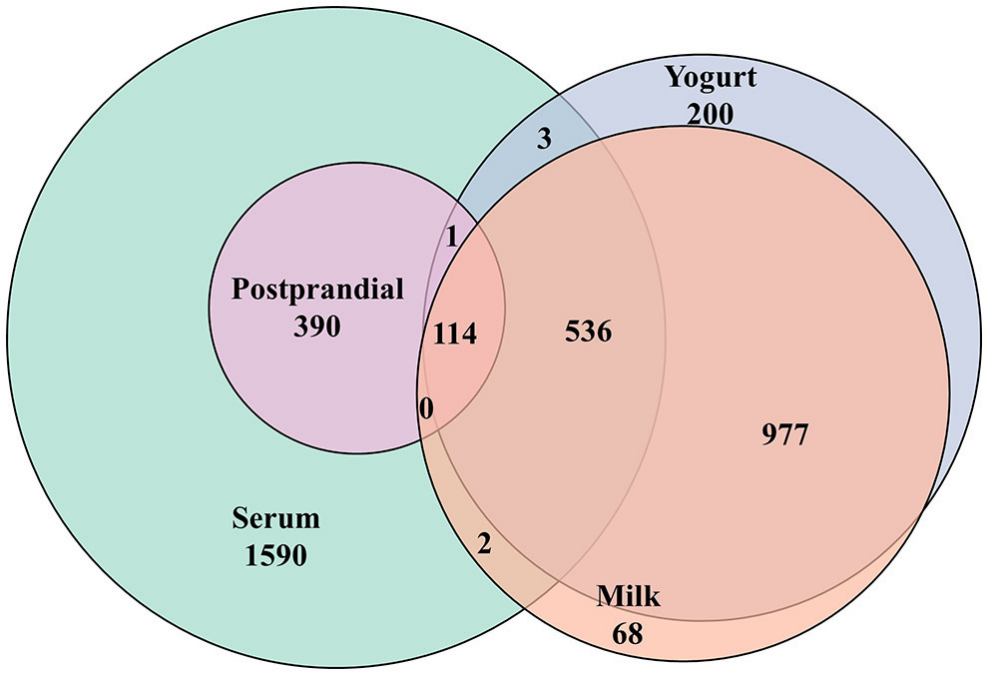
Venn diagram summarizing the common metabolic features (LC-MS) detected in milk, yogurt, and the human serum before (fasting) and after (postprandial) the intake these two dairy products.

A total of 1,901 features were detected in the dairy products used in the present study (milk: 1,697, yogurt: 1,831) (Figure 2). Among them, 1,627 features were detected in both milk and yogurt, which accounted for 85.6% of total features. Thousand hundred and eight features showed significant quantitative differences between the two products: among these 520 were higher in milk and 488 were higher in yogurt (FDR < 0.1).

### Global Characteristics of the Postprandial Features

A total of 2,565 features were detected by untargeted LC-MS analyses in the serum samples (see Figure 2). Among them, 505 features showed significant responses during the postprandial phase, which accounted for 19.7% of all detected features. Among them, 115 features showed an overlap with the metabolome of milk or yogurt. Among the 505 postprandial features, 24 features showed significant differences in iAUCs after milk and yogurt intake (FDR <0.1, 83 features by *P* < 0.05), 31 features between the YA and the OA groups (FDR < 0.1, 109 features by *P* < 0.05). Also, 15 features showed an interaction between the type of dairy product and age (FDR < 0.1, 70 metabolites by *P* < 0.05).

**Figure 3:**
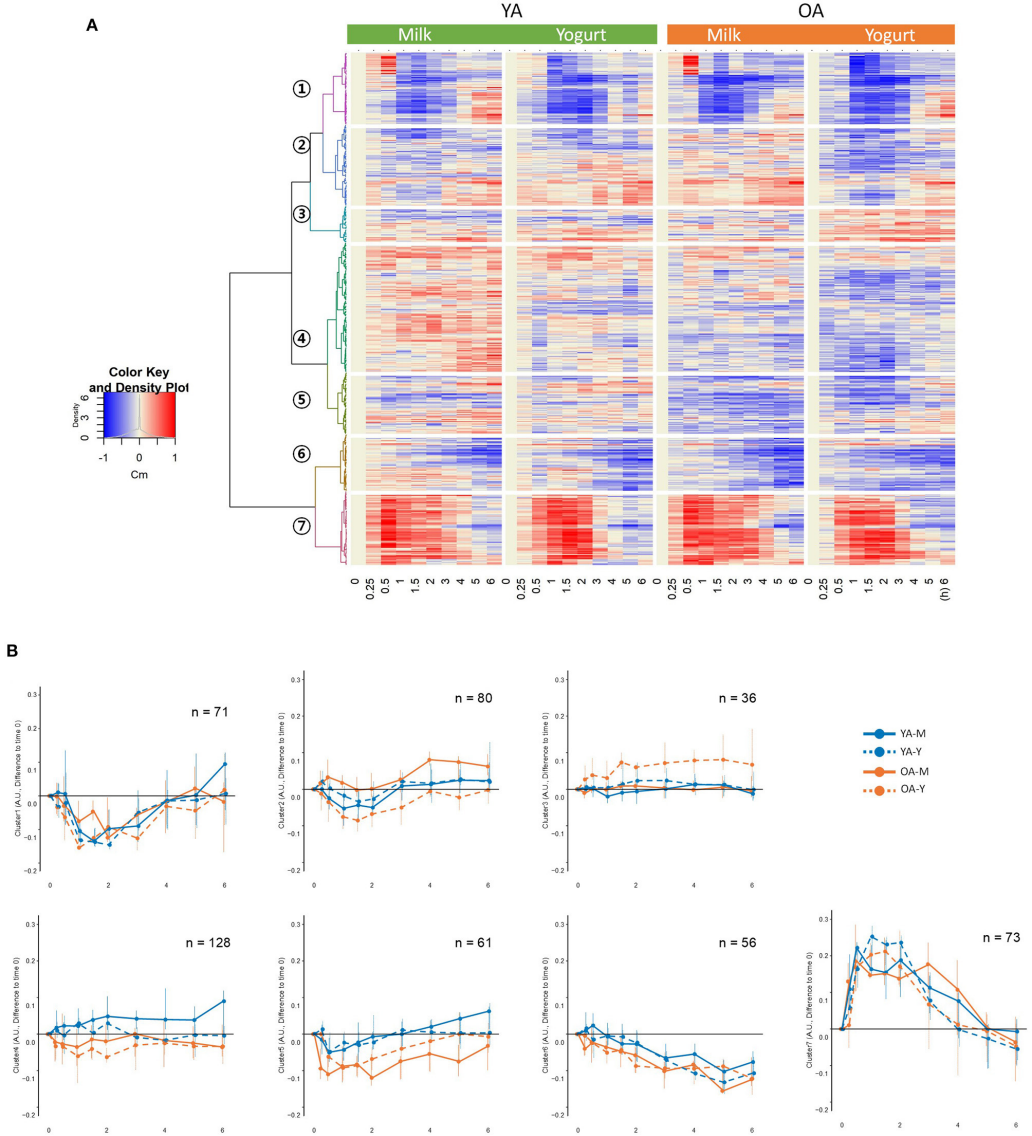
**(A)** Hierarchical clustering analyses on the 505 metabolic features showing significant responses during the postprandial phase. **(B)** postprandial representation of the mean values for the metabolic features belonging to the seven identified clusters. YA-M, young adults-milk intake; YA-Y, young adults-yogurt intake; OA-M, older adults-milk intake; OA-Y, older adults-yogurt intake.

Hierarchical clustering analyses on the postprandial features presented seven clusters separated according to the kinetics of their postprandial responses after milk or yogurt intake (Figure 3A). While clusters 2–5 showed relatively minor kinetic changes, stronger postprandial changes were observed with decreased features in cluster 1 (71 features) and increased features in cluster 7 (73 features) (Figure 3B). The global postprandial responses of clusters 1 and 7 appeared visually similar between the milk and yogurt intake as well as between the YA and OA groups. The features in these two clusters were therefore further investigated kinetically (see below).

### Discriminant Postprandial Metabolites

**Figure 4:**
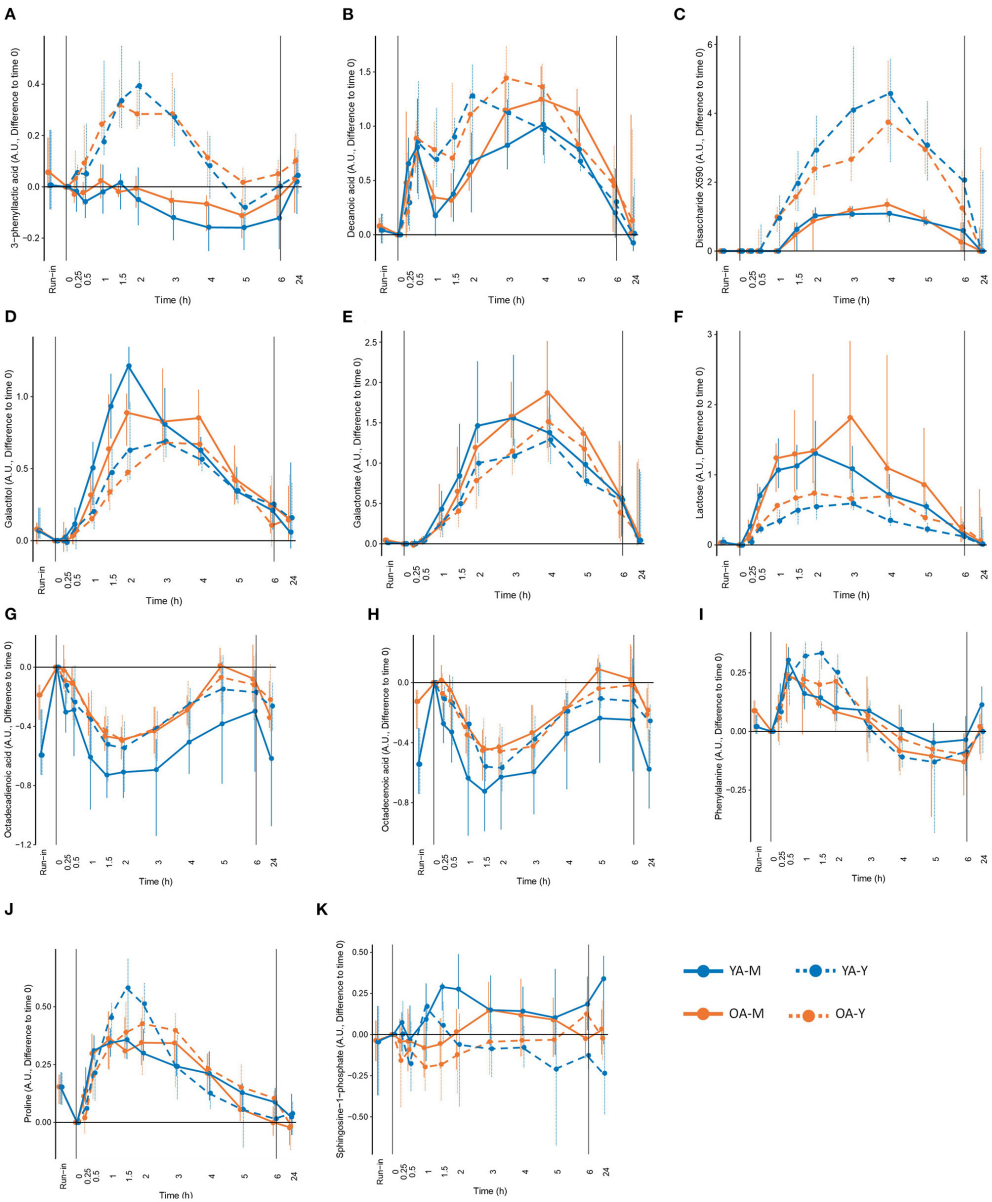
Selected metabolites that could be interesting as candidate biomarkers of the intake of milk (M), yogurt (Y), or dairy in the young (YA) and the older (OA) men, based on the significant modification during the exclusion of dairy intake (run-in), different concentration in milk and yogurt, significant differences in postprandial responses between milk and yogurt intake, and modification in fasting levels after 24h of dairy intake. **(A)** 3-phenyllactic acid, **(B)** decanoic acid, **(C)** disaccharide X590, **(D)** galactitol, **(E)** galactonate, **(F)** lactose, **(G)** octadecadienoic acid, **(H)** octadecenoic acid, **(I)** phenylalanine, **(J)** roline, and **(K)** sphingosine-1-phhosphate. See Table 1 for detailed information on differences following the run-in period and 24h following the dairy intake.

Among the 63 postprandial metabolites identified by LC-MS or GC-MS in this study, 31 metabolites showed significant differences in their postprandial responses by the type of dairy product, by age, and/or by the interaction between the type of dairy products and age (Table 1). According to HMDB, 11 metabolites were classified as lipids, fatty acids and derivatives, 10 metabolites were classified as amino acids and derivatives, 6 metabolites were classified as carbohydrates and derivatives, and 4 metabolites belonged to other classes of molecules. In addition to the postprandial responses, Table 1 also shows whether the fasting values of these metabolites were changed during the run-in phase, whether the postprandial changes were still visible after 24 h following the dairy intake and whether these metabolites were present in different concentrations in the dairy products themselves (milk and yogurt). Details on the postprandial kinetics of 11 metabolites discriminating milk, yogurt, or dairy intake are showed for each of the four conditions (YA-M, YA-Y, OA-M, OA-Y) in Figure 4.

#### Lipids, Fatty Acids, and Derivatives

Among the 11 metabolites classified as ‘lipid, fatty acids and derivatives', 5 had significant positive iAUCs (2-hydroxybutyric acid, decanoic acid, dodecanoic acid, myristic acid, glycocholic acid) and 6 had significant negative iAUCs (3-dehydroxycarnitine, decanoylcarnitine, dodecanoylcarnitine, octadecenoic acid, octadecadienoic acid). The iAUCs of 7 of them showed a significant effect of age (3-dehydroxycarnitine, decanoylcarnitine), product (decanoic acid, dodecanoic acid, sphingosine-1-phosphate, 3-dehydroxycarnitine), or an interaction (2-hydroxybutyric acid, glycocholic acid).

The negative iAUCs of 3-dehydroxycarnitine and decanoylcarnitine tended to be higher in the OA group than for the YA group (*P* 0.06 and *P* 0.065, respectively). This effect remained significant after milk and yogurt intake when both dairy groups were analyzed separately for decanoylcarnitine but only after milk intake for 3-dehydroxycarnitine.

The positive iAUCs of decanoic acid were significantly higher after yogurt intake than after milk intake (*P* 0.006). This effect remained significant in the OA group when both age groups were analyzed separately. Interestingly, the run-in phase reduced the levels of decanoic acid, although this effect was only significant in the OA group. The positive iAUCs of dodecanoic acid were also significantly higher after yogurt intake than after milk intake (*P* 0.003). This effect, however, were no longer significant when both age groups were analyzed separately. The iAUCs of sphingosine-1-phosphate were reduced after yogurt compared to milk intake. This effect remained significant in the YA group when both age groups were analyzed separately. The negative iAUCs of 3-dehydroxycarnitine were significantly lower after yogurt intake than after milk intake (*P* 0.039). This effect also remained significant in the YA group when both age groups were analyzed separately.

Interactions between age and product effects were observed for 2-hydroxybutyric acid and glycocholic acid. The iAUC of 2-hydroxybutyric was significantly higher after yogurt intake than milk intake in the YA group, but not in the OA group, whereas, compared to the YA group, the OA group presented higher iAUC after milk intake, but not after yogurt intake. Similarly, the iAUC of glycocholic acid was significantly higher after yogurt intake than milk intake in the YA group, but not in the OA group, whereas, compared to the YA group, the OA group presented lower iAUC after yogurt intake, but not after milk intake.

#### Amino Acids and Derivatives

Among the 10 metabolites classified as ‘amino acids and derivatives', 7 had a significant positive iAUCs (aspartic acid, lysine, ornithine, phenylalanine, proline, tyrosine, N-methyl proline), 1 had negative iAUCs (3-methylhistidine), and 1 had mixed iAUCs (3-phenyllactic acid). Among the 10 metabolites, the iAUCs of 3 of them showed a significant effect of age (tyrosine), product (aspartic acid, 3-phenyllactic acid), or an interaction (3-methylhistidine).

The positive iAUCs of tyrosine were more pronounced in the OA group than for the YA group although this effect was no longer significant when both dairy groups were analyzed separately.

The positive iAUCs of aspartic acid were less pronounced after yogurt intake than after milk intake, although this effect was no longer significant when both age groups were analyzed separately. The iAUCs of 3-phenyllactic acid were significantly increased after yogurt intake compared to milk intake. This effect remained significant when both age groups were analyzed separately. Interestingly, this metabolite was also much more abundant in the yogurt product than in milk (FDR <0.001). An interaction effect was demonstrated for 3-methylhistidine (*P* 0.031), the negative iAUC of this amino acid derivative being significantly reduced in the OA group compared to the YA group after milk intake but not after yogurt intake.

#### Carbohydrates and Derivatives

Among the 6 metabolites classified as ‘carbohydrates and derivatives', 5 had significant positive iAUCs (lactose, galactose, galactitol, galactonate, X0590) and 1 had significant negative iAUCs (glycerol). Among the 6 metabolites, the iAUC of 4 of them showed a significant effect of age (lactose) or product (lactose, galactitol, galactonate, X0590).

The positive iAUC of lactose, galactitol, and galactonate were all lower after yogurt intake than milk intake. This effect was retained for both lactose and galactonate when the YA and OA groups were analyzed separately, whereas it only reached statistical significance in the YA group for galactitol. Interestingly, lactose was significantly decreased during the semi-controlled phase in the YA and OA group, galactitol was decreased in the YA group, and galactonate was decreased in the OA group. In addition to a product effect, postprandial lactose demonstrated an age effect, the iAUC after dairy intake being higher in the OA than the YA group. This effect retained significance after yogurt intake, but not milk intake, when the intake of both products was analyzed separately. 24 h after the dairy challenges, increased fasting levels were observed in the OA-M and OA-Y conditions for lactose, YA-Y and OA-Y conditions for galactitol, and all four conditions for galactonate.

In contrast to the above metabolites derived from lactose, the positive iAUCs of the unidentified disaccharide X0590 were significantly higher after yogurt intake in both the YA and AO groups. This molecule was still present at higher concentration 24 h after the dairy challenge. Furthermore, it was present in higher concentrations in the yogurt product than in milk.

#### Other Organic Compounds

Among the 4 metabolites classified as ‘other organic compounds', 2 had significant positive iAUCs (epinephrine, citrate). Among the 4 metabolites, the iAUCs of 2 of them showed a significant effect of age (isocitric acid, inosine).

The iAUCs of isocitric were less pronounced in the OA group than for the YA group although this effect was no longer significant when both dairy groups were analyzed separately. Inconsistent results were observed when this molecule was evaluated during the run-in phase as well as 24 h after the dairy challenge. The iAUCs of inosine were significantly lower in the OA group than in the YA group (*P* 0.045). This effect remained significant when both dairy groups were analyzed separately.

### Discriminating Metabolites by Kinetic Analyses

The kinetic parameters C_max_ and t_max_, for the metabolites with positive postprandial responses in cluster 7 (71 features), and C_min_ and t_min_, for the metabolites with negative postprandial responses in cluster 1 (73 features), were calculated. As presented above, among the 31 metabolites identified in Table 1, 15 of them did not present any significant differences by product, by age, and/or by interaction in their postprandial iAUC. However, by investigating kinetic parameters, 14 of these 15 metabolites revealed age, product, and/or interaction effects.

Four metabolites in the ‘lipid, fatty acid, and derivatives' group showed an age and/or product effect on their C_max_ (C_min_) not revealed by iAUC analyses. The C_max_ of myristic acids was higher in the OA group then in the YA group. This effect was retained for milk intake when the YA and OA groups were analyzed separately. The C_min_ of octadecenoic acid and octadecadienoic acid were smaller after yogurt intake compared to milk intake, although the product effect was only retained for octadecadienoic acid in the YA group when the YA and OA groups were analyzed separately. Of note, 24 h after the intake of both dairy products, significantly decreased fasting values of octadecenoic acid were observed, compared to the fasting values in all test conditions compared to the baseline before each dairy challenge (YA-M, YA-Y, OA-M, OA-Y). A similar observation was made for octadecadienoic acid in 3 test conditions (YA-M, YA-Y, OA-M). Moreover, the fasting levels of both fatty acids (octadecenoic and octadecadienoic) were significantly increased by the end of the semi-controlled phase in both the YA and OA group. Dodecanoylcarnitine showed both age and product effect on the C_min_ but only the age effect was retained when milk and yogurt intake were analyzed separately, presenting a more pronounced negative C_min_ in the OA group compared to the YA group after milk intake.

Six metabolites in ‘amino acids and derivatives' group showed age (phenylalanine, threonine), product (lysine, ornithine, phenylalanine, proline, N-methyl proline, threonine), as well as interaction effects (lysine, ornithine, proline, N-methyl proline, threonine) on their C_max_ and/or t_max_ not revealed by iAUC analyses. The t_max_ of phenylalanine was delayed in the OA group compared to the YA group and this effect remained significant after milk intake when the products were analyzed separately. The C_max_ of threonine was lower in the OA compared to the YA group although this effect was only seen after the intake of yogurt. Lysine, phenylalanine, N-methyl proline, and threonine had delayed t_max_ after yogurt intake compared to milk. However, only the delayed t_max_ of phenylalanine retained significance when the YA and OA groups were analyzed separately. The product effects identified for the C_max_ of ornithine and proline were less clear. Interestingly, the fasting levels of phenylalanine in the OA group and proline in both age groups significantly decreased during the run-in period. In addition, interaction effects were observed in the C_max_ of lysine, ornithine, proline, N-methyl proline, and threonine, which will not be describe in further details here.

Two metabolites in ‘carbohydrates and derivatives' group showed product (galactose, glycerol) effects on their C_max_ (C_min_) not revealed by iAUC analyses. The lower C_max_ of galactose observed after yogurt intake compared to the milk intake in both age groups, indicating similar effects as observed with the iAUC of lactose and its derivatives galactitol and galactonate. Also, the C_min_ of glycerol was smaller after yogurt intake compared to the milk intake, although significance was lost when the age groups were analyzed separately.

Two metabolites in ‘other organic compounds' group showed product (epinephrine, citrate) and interaction effect (epinephrine) on their t_max_ not revealed by iAUC analyses Although the t_max_ of both metabolites were delayed after yogurt intake compared to milk intake, the product effect only retained significance for epinephrine in YA group when the age groups were analyzed separately.

## Discussion

In the present intervention study, we applied a metabolomics-based strategy (LC-MS, GC-MS) to explore the postprandial dynamics of serum metabolites following the intake of two specific dairy products (milk and yogurt) to elucidate if some of them could act as specific candidate BFI of these foods products. We further explored if the postprandial responses of these candidate biomarkers were different in older individuals when compared to younger adult subjects. We found that, beyond their potential role as BFI, the metabolomics profiles were influenced by the type of ingested dairy product. Although the impact of dairy intake was predominant on that of aging, interestingly, for some of these parameters, interactions between the product type and the age group were observed, suggesting that physiological changes induced by the aging process, e.g., metabolic flexibility (35), could differently influence the postprandial response to these two dairy products. We further identified two clusters of metabolites, measured by untargeted LC-MS, showing both positive (cluster 7) or negative (cluster 1) postprandial responses to the product intake, together with selected metabolites measured by targeted GC-MS, which were discussed as potential dairy intake biomarkers and/or in the context of their physiological relevance.

### Discriminant Metabolites Between Milk and Yogurt Intake

A total of 25 identified metabolites showed different postprandial responses between milk and yogurt intake, which can be explained in part by the changes in the product composition, induced by the fermentation. Firstly, lactose levels in yogurt decrease during fermentation due to its hydrolysis into glucose and galactose by the lactic acid bacteria in yogurt. Consequently, we found lower serum levels of lactose and its derivatives such as galactonate and galactitol after yogurt intake compared to the milk both in young and older participants. Galactonate is produced from galactose oxidization by galactose dehydrogenase in the liver while galactitol is formed by aldose reductase action (36) and both have been previously proposed as candidate BFI, at least in young adult subjects (16, 37). Interestingly, galactonate was detected in yogurt but not in milk, suggesting that lactic acid bacteria possess enzymatic activity able to metabolize galactose into galactonate. Galactonate can be used by lactic acid for their growth (38) but evidence appears to be lacking for the bacterial production of this molecule. Secondly, various bacterial products are synthesized and released into the yogurt during the fermentation process. This is the case of 3-phenyllactic acid, a bacterial product derived from phenylpyruvate by NADH-dependent lactate dehydrogenase (39), that was more abundant in our yogurt samples. Accordingly, and in line with previous studies in which its potential role as yogurt and cheese intake biomarker was proposed (16, 17, 40), we also observed that 3-phenyllactic acid, was particularly elevated after yogurt consumption. However, given that this metabolite is also found in other fermented foods (sauerkraut, honey…) and added to several industrial products as a biological preservative (41), it seems more reasonable to consider it as able to sign the consumption of fermented foods overall, rather than as a specific biomarker of cheese or yogurt intake. Various exopolysaccharides and galacto-oligosaccharides are also produced by metabolic activities of yogurt culture during the fermentation process (42). In the present study, the detection of a not yet identified disaccharide with a strong discrimination capacity between milk and yogurt intake could be likely explained by the complex mixture of saccharides in the yogurt (42). Once identified, this molecule could be an interesting candidate biomarker of yogurt intake, which is further supported by the fact that it was also highly abundant in the yogurt itself. Although yogurt fermentation results in relatively small changes in fat composition (43), we also found significantly higher iAUCs of sphingosine-1-phosphate after milk intake. Sphingosine-1-phosphate, a metabolite of sphingolipids catabolism, has been implicated as a modulator of various physiologic processes such as cell survival and proliferation (44) and inflammatory responses (45). While in our study sphingosine-1-phosphate was below the detection limit in our products, previous studies found that the content of sphingolipids is 2-fold higher in milk than in yogurt (46) and free sphingoid bases are 100-fold higher in non-fat dry milk compared to yogurt (47), supporting sphingosine-1-phosphate as a candidate biomarker of milk intake. Fermentation can also increase free amino acids content due to the bacterial peptidases from lactic acid bacteria (48), which may explain the higher abundance of plasma proline and phenylalanine, after the yogurt intake compared to the milk intake, and in accordance with their higher abundance in our yogurt samples. Although these findings are interesting regarding the contribution of milk fermentation to the amounts of postprandial amino acids available for systemic metabolism, the unspecific nature of amino acids renders them unsuitable as BFIs. Another important modification induced by the fermentation process is the transformation of the liquid matrix of milk into the semi-solid matrix of yogurt, due to the reduction in the pH, that increases the casein-casein attractions, and favors the formation of a three-dimensional network (49), leading to the increased viscosity of yogurt. In our study, this matrix effect could indeed be a major contributor to the lower C_max_ and the delayed t_max_ of several circulating metabolites that were trapped in the structure (amino acids, lactose, fatty acids…).

Confirming the validity of some of the discriminant metabolites described above as reliable biomarkers of milk, yogurt, or dairy intake is challenging, in particular because their specificity for selected dairy products, such as milk or yogurt, even dairy products in general, is rarely given (50). In addition, the human metabolome partially overlaps with those of dairy products, with 650 common metabolic features in our study. For instance, amino acids and fatty acids are evidently abundant in numerous other foods. Concerning lactose, although this metabolite is also present in certain drugs as an excipient (51), urinary lactose and galactose were associated with dairy product consumption in the Karmen cohort (52), indicating that the confounding effects of lactose in drugs is limited. Nevertheless, a strength of the present study is the strictly dietary control exerted during the run-in period, which allowed to reinforce the potential role of several discriminant metabolites identified postprandially as candidate biomarkers of dairy intake. Indeed, significantly reduced fasting levels of lactose, galactonate, and galactitol were observed during the run-in period. Also, the acute intake of milk or yogurt resulted in increased levels of lactose, galactose, and galactitol that were still observed 24 h after the dairy challenge, indicating that these markers may be useful beyond the acute postprandial phase. Although amino acids such as proline and phenylalanine are not specific to dairy products, our findings support the use of fermented dairy products for improving the nutritional delivery of amino acids, in line with previous reports (53). Another interesting observation could be made for several fatty acids showing negative postprandial responses to milk and yogurt intake, i.e., octadecenoic acid (C18:1) and octadecadienoic acid (C18:2). Not only the levels of these metabolites 24 h after the dairy challenge are still low, but their levels actually increased during the run-in period. The postprandial behavior of these lipids is complex as likely influenced by the fermentation of milk, processing by the gut microbiota and systemic metabolism (26). A more detailed discussion of these molecules would request that their molecular structure be precisely determined by high resolution GC-FID (26).

On the other hand, some of the candidate biomarkers of milk, yogurt, or dairy intake suggested previously in the literature could not be verified in the present study, including indole derivatives (40), glycocholic acid (40) and heptadecanoic acid (C17:0) (18). These results could be explained by specificities linked to the microbiota of the study population (here both adult and elderly), the nature and production process of the consumed products, and the analytical sensitivity of the metabolomics analyses, which deserve further attention in future interventions. In particular, we found that the commercial starter culture had low levels of *L. delbrueckii* subsp. *bulgaricus* and was below the detection limit in yogurt in agreement with the absence of D-lactate, which is not produced by *S. thermophilus* (54). *L bulgaricus* is a proteolytic species (55). Also, several amino acid biosynthetic pathways are induced in *S. thermophilus*, which *L. bulgaricus* does not possess (54). As mentioned above, the lack of metabolic activity of *L. bulgaricus* is a desired trait in mild acidifying yogurts. However, this characteristic might have influenced the metabolome of the yogurt and, consequently, the composition of the postprandial serum, eventually by diminishing the number of metabolites differentiating the postprandial response of the study participants to milk and yogurt.

### Altered Metabolism in the Older Population

The physiological modifications induced by age also influenced the postprandial responses of various metabolites to dairy intake. For instance, the delayed t_max_ of lactose and galactonate in the OA compared to the YA group might be explained by the prevalent intestinal lactase deficiency and the impaired absorptive capacity in older adults (56). In addition, the less efficient production of primary bile acids in the liver (35) and the modified gut microbiota composition during aging (57) could explain the lower levels of glycocholic acid observed in the OA group in the present study. However, our previous study with the same study individuals revealed that there were no significant differences in gut microbiota profile between the YA and the OA groups (26), suggesting the less efficient production of primary bile acids in the liver could be the major factor of the lower glycocholic acid levels in the OA group. Lipid metabolism was particularly influenced by age, presenting delayed t_max_ of dodecanoic acid (C12:0), myristic acid (C14:0), and acylcarnitines in the OA group. Acylcarnitines are essential compounds for the fatty acid transportation into the mitochondria for subsequent beta-oxidation. Therefore, the lower levels of acylcarnitines could reflect in part the higher levels of circulating fatty acids, likely as a consequence of a reduction in fatty acid oxidation, to which a less efficient metabolic capacity and reduced muscle mass during aging could contribute (58). Further support for this particular profile could be supplied by a reduced lipolytic activity and sensitivity to the anti-lipolytic effect of insulin reported in the older population (59), resulting in a slower clearance of fatty acids.

### Interaction Between Dairy Products and Aging

Among the metabolites discriminating between milk and yogurt intake, lactose, proline, ornithine, and epinephrine presented different profiles depending on the age group. Overall, delayed t_max_ and increased C_max_ were more often observed after the intake of milk in the OA group compared to the YA group. A recent study compared gastric digestion of milk proteins in adult and elderly *in vitro* digestion models and found that the elderly model showed significantly delayed digestion with aggregated milk proteins (60). This study is thus in line with our results showing that milk transformation (i.e., fermentation to yogurt) differently impacts its processing by the gastrointestinal tract as a function of age. In addition, a prospective study found that the secretion of pepsin in the elderly aged 65–98 years decreased by ~40% compared to young adults (18–34 years old) (61), which could limit the digestive proteolysis. Therefore, the digestion of coagulated caseins in the stomach could be less efficient in the older population compared to the young.

Overall, although our study presents several strengths on the BFI research strategy, including the exploration of the postprandial period and the uncommon strict dietary control exerted during the run-in period, it also has several limitations. For instance, some of changes observed on several metabolites seem to reflect the global physiological response to the meal rather than specifically the dairy products intake. Moreover, additional test arms (dose response, isocaloric non-dairy group, extension of the study to female participants) should be considered in the future to further confirm the specificity of the dairy candidate of BFI presented here.

## Conclusion

Due to the specific physiological modifications accompanying the aging process, the differences observed in our study in the behavior of several metabolites after milk and yogurt intake were likely impacted by the age variable in the OA group. Nonetheless, the differences in postprandial behavior of the metabolites reported here between the YA and OA groups remain qualitative in nature and, for most of them, subtle. This observation indicates that the molecules selected for further validation as biomarkers of dairy product intake could be used in a broad range of consumers across the lifespan. On the other hand, the age-dependent differences in their behavior observed in this study should be considered, in particular when investigating necessary steps for BFIs validation, e.g., their dose-response (62).

To conclude, our global analysis of the postprandial serum metabolome in response to dairy products intake in healthy men revealed a robust response organized in different kinetic clusters. Although the global response of the postprandial metabolome (several clusters) was rather independent on the age group, several metabolites showed changes likely explained by a healthy aging process, including delays in the postprandial response of several lipids, galactonate and lactose as well as a lower glycocholic acid response. The postprandial metabolome was different following the intake of milk and yogurt. Among the identified metabolites being discriminant between milk and yogurt intake, some of them confirmed previous studies, including lactose, galactonate, and galactitol (higher after milk intake) or 3-phenyllactic acid (higher after yogurt intake, but rather considered as a biomarker of fermented food). Other metabolites, like sphingosine-1-phopshate and an unidentified disaccharide, should be further studied for validation as potential biomarkers of milk and yogurt intake, respectively.

## Data Availability Statement

The original contributions presented in the study are publicly available. This data can be found here: MetaboLights, MTBLS174, https://www.ebi.ac.uk/metabolights/MTBLS174/descriptors.

## Ethics Statement

The studies involving human participants were reviewed and approved by Ethical Committee of Personal Protection Ile de France IV (protocol code: 2017-A02879-44). The patients/participants provided their written informed consent to participate in this study.

## Author Contributions

JK, CM, DD, SP, and GV: conceptualization. JK: data curation, visualization, and writing—original draft preparation. JK and JD: methodology. JK, CB, RB, HS, and UB: formal analysis. JK, SP, UB, and GV: writing—review and editing. JK, CM, SP, and GV: project administration. DD, SP, and GV: funding acquisition. All authors have read and agreed to the published version of the manuscript.

## Funding

This research was funded by French Dairy Interbranch Organization, Agroscope and INRAE.

## Conflict of Interest

JK and CM are employees of French Dairy Interbranch Organization. The authors declare that this study received funding from CNIEL. The funder had the following involvement in the study: participation to the conceptualization of the study and administration of the project.

## Publisher's Note

All claims expressed in this article are solely those of the authors and do not necessarily represent those of their affiliated organizations, or those of the publisher, the editors and the reviewers. Any product that may be evaluated in this article, or claim that may be made by its manufacturer, is not guaranteed or endorsed by the publisher.
