# Supplementary material for: Postprandial Responses on Serum Metabolome to Milk and Yogurt Intake in Young and Older Men
Source: Front Nutr. 2022 May 4;9:851931. doi: 10.3389/fnut.2022.851931 (PMC9115859; doi:10.3389/fnut.2022.851931)
Supplement: Supplementary file 1 [file Data_Sheet_1.docx]

**SUPPLEMENTARY DATA**

**Supplementary Figure 1**. Flow diagram of the study.


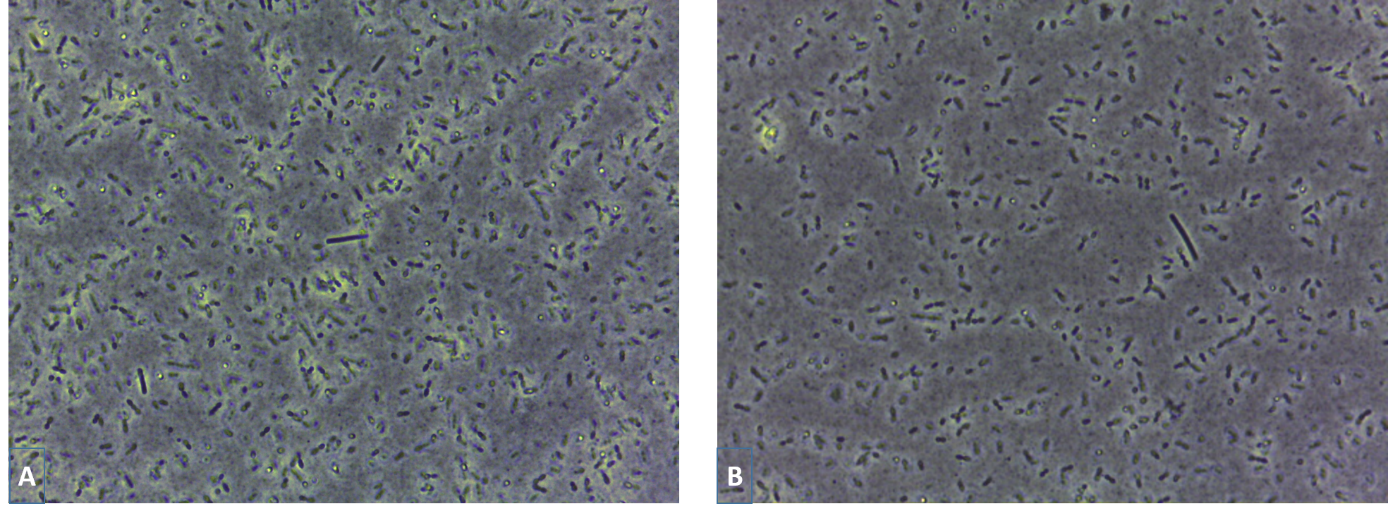


**Supplementary Figure 2:** A) Microscopic image of freeze-dried powder in NaCl solution to achieve a 1000x concentration compared to yogurt inoculation. B). Microscopic image of yogurt after incubation at 43 °C to reach pH 4.6**.** No sample treatment was applied. Both images were taken with 1000x magnification. The microscopic fields were selected for the presence of at least one rod.

**Supplementary Table 1.** Composition of dairy products consumed UHT milk and yogurt (n=5)

| **Nutrient** | **UHT Milk** | **Yogurt** | ***P*-value** |
| --- | --- | --- | --- |
| Energy ^a^, kcal/100g | 65.11 (64.05, 66.61) | 65.64 (63.74, 67.31) | 0.836 |
| Protein, g/100g | 3.26 (3.21, 3.38) | 3.37 (3.29, 3.44) | 0.057 |
| Casein, g/100g | 2.63 (2.57, 2.73) | 2.71 (2.68, 2.78) | **0.036** |
| Whey proteins, g/100g | 0.64 (0.53, 0.70) | 0.63 (0.56, 0.70) | 0.863 |
| Non protein N, g/100g | 0.00 (0.00, 0.01) | 0.00 (0.00, 0.00) | **0.003** |
| Fat, g/100g | 3.70 (3.68, 3.74) | 3.85 (3.81, 3.91) | **< 0.001** |
| Carbohydrate, g/100g | 4.78 (4.65, 4.88) | 3.86 (3.38, 3.97) | **< 0.001** |
| Lactose, g/100g | 4.78 (4.64, 4.85) | 3.08 (2.98, 3.13) | **< 0.001** |
| Glucose, g/100g | 0.01 (0.00, 0.01) | 0.10 (0.08, 0.12) | **< 0.001** |
| Galactose, g/100g | 0.01 (0.00, 0.01) | 0.77 (0.29, 0.84) | **< 0.001** |
| Lactic acid L-, g/100g | n.d. | 0.84 (0.82, 0.87) | **< 0.001** |
| Lactic acid D+, g/100g | n.d. | n.d. | **-** |
| Dry matter, g/100g | 12.64 (12.58, 12.78) | 12.87 (12.82, 12.99) | **< 0.001** |
| Ash, g/100g | 0.74 (0.72, 0.76) | 0.74 (0.74, 0.75) | 0.833 |

Each value is the median (Interquartile range (IQR)). ^a^ Calculated by Atwater system. ^a^ at the end of fermentation. *Streptococcus thermophilus* was present in the five batches in the range of 6.0-8.2 E+08 CFU/g; *Lactobacillus delbrueckii* spp. *bulgaricus* was below the detection limit (< 1 CFU/g) in all batches. The absence of D-lactic acid in yoghurt is due to the lack of growth of *Lactobacillus delbrueckii spp. bulgaricus*. n.d.: not detected
